# Proton Activity as a Promoter for the CO_2_ Electroreduction to Formic Acid in Acidic Media

**DOI:** 10.1002/cssc.70906

**Published:** 2026-07-23

**Authors:** Thomas Mairegger, Christoph Griesser, Philipp Stadler, Alexander Beck, Julia Kunze‐Liebhäuser

**Affiliations:** ^1^ Department of Physical Chemistry University of Innsbruck Innsbruck Austria; ^2^ Net Zero Emission Labs GmbH Rohrdorf Germany

**Keywords:** carbonate, electrochemical reduction of carbon dioxide, electrochemistry, electrosynthesis, formic acid, kinetics, proton

## Abstract

The electrochemical carbon dioxide reduction reaction (CO_2_RR) in acidic electrolytes addresses the critical issue of carbonate formation but is intrinsically limited by the parasitic hydrogen evolution reaction (HER). While prevailing strategies aim at suppressing the HER by depleting interfacial proton concentration, this approach is detrimental for products whose formation kinetics are proton‐dependent. We demonstrate that for the electrosynthesis of formic acid (FA), the maintenance of high interfacial proton activity is not merely a challenge but a requirement for higher FA formation rates. We show that, rather than arising from suppression of the competing HER, FA synthesis is directly coupled to proton availability and is strongly enhanced at higher proton activities. Our findings establish that an efficient acidic CO_2_RR to FA requires a distinct design paradigm, which balances the kinetic competition between HER and CO_2_RR while ensuring sufficient proton flux to drive the kinetics of FA formation.

## Graphical abstract

Challenging the standard of proton suppression, this research identifies high proton activity as a crucial driver for high formic acid production rates. The resulting design paradigm highlights that balancing proton and CO_2_ concentrations is the key to achieving high formation rates and selectivity in acidic electrolytes.

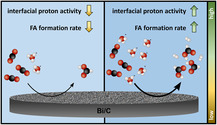

## 1 Introduction

The electrochemical CO_2_ reduction reaction (CO_2_RR) offers a viable pathway to address global warming and advance a circular carbon economy [1]. Generally, the CO_2_RR can generate various products ranging from hydrocarbons to oxygenates [2, 3, 4]. However, the industrial sector currently targets C1 species, specifically formic acid (FA) and carbon monoxide (CO), due to the high Faradaic efficiencies (FE) and current densities attainable with existing electrocatalysts [1, 2, 3, 5, 6]. Current research efforts predominantly prioritize alkaline environments and anion exchange membranes (AEMs), a strategy largely driven by the need to mitigate the parasitic hydrogen evolution reaction (HER), which exhibits intrinsically sluggish kinetics in high‐pH media. Despite the suppression of the HER at high pH, this approach is fundamentally limited by the thermodynamic instability of CO_2_ in alkaline electrolytes, where carbonate formation and salt precipitation compromise electrocatalytic performance [7, 8, 9, 10]. Consequently, shifting to acidic conditions offers a decisive advantage: It not only circumvents the stability bottlenecks but also facilitates the direct production of liquid FA (pK_A_ = 3.75), thereby simplifying downstream separation [8, 10, 11]. Therefore, elucidating catalytic pathways that effectively suppress the HER in acidic electrolytes is important for advancing industrial CO_2_ electroreduction to FA. In this context, bismuth (Bi)‐based materials emerge as premier candidates, distinguished by their intrinsic catalytic activity toward FA synthesis and their characteristically high kinetic overpotential for the competing HER [12, 13, 14, 15, 16, 17].

To suppress the parasitic HER, investigations frequently focus on mildly acidic electrolytes (pH 3) [8, 18, 19]. This approach serves as a strategic compromise: It sufficiently limits the effective proton concentration, aka proton activity, to mitigate the HER dominance, and yet remains below the pK_a_ of FA to ensure the direct formation of the protonated liquid product rather than formate [8, 19]. Furthermore, the prevailing consensus in current acidic CO_2_RR literature predominantly focuses on maximizing FE by deliberately restricting interfacial proton availability. This is typically achieved through strategies such as utilizing specific alkali metal cations to repel proton migration or engineering hydrophobic microenvironments [4, 8, 19, 20, 21, 22]. Recent mechanistic investigations, mainly focused on the CO_2_ reduction to CO, have established a “proton depletion” strategy to mitigate the HER in acidic media [4, 9, 20, 21]. In these systems, the rate‐determining step (RDS) for CO formation, i.e., the electron transfer to adsorbed CO_2_, is decoupled from the proton concentration prevailing at the electrode surface, allowing water to serve as the proton donor under mass transport–limited conditions [21]. Consequently, the locally generated hydroxide ions (OH^−^) neutralize the incoming proton flux, when the CO_2_ reduction rate is sufficiently high, and effectively create a suppression zone that inhibits the HER without stifling CO production.

While these “proton depletion” strategies successfully enhance selectivity, our study demonstrates that they limit the CO_2_RR rate to FA. Because restricting proton availability creates a kinetic bottleneck, we show that elevated proton activity, encompassing both effective proton concentration and mass transport, is crucial for maximizing the absolute FA production rate, as protons serve as a direct reactant. (Equation I).

(I) $${\mathrm{CO}}_{2}+2H^{+}+2e^{-}\to \text{HCOOH}$$

## 2 Experimental Section

### 2.1 Synthesis of Nanoporous Carbon‐Supported Bismuth (Bi) and Preparation of Bi/C Ink

In all experiments, carbon‐supported Bi nanoparticles (Bi/C) mixed with a Nafion ionomer (D2021 Nafion solution, Ion Power) in a 70/30 wt% ratio were used as electrocatalyst ink. The ink was drop‐casted onto a glassy carbon support, resulting in a catalyst loading of 0.26 mg/cm^2^. Detailed procedures regarding the nanoparticle synthesis and ink preparation, along with ex situ characterizations (XRD, TEM, and XPS before and after electrolysis), are reported in our previous publication [18].

### 2.2 Electrochemical Characterization

Linear sweep voltammograms (LSVs) were carried out at room temperature in a classical three‐electrode setup, using a SP‐150e potentiostat (BioLogic). The all glass three‐electrode cell consisted of a Bi/C working electrode (WE), a silver/silver chloride (Ag/AgCl) reference electrode (RE), and a carbon rod counter electrode (CE). The electrolyte was 0.1 M K_2_SO_4_, adjusted to different pHs using H_2_SO_4_. The electrolyte pH remained constant at the respective desired value, even after purging the solution with CO_2_ for 1 h. All reported potentials were converted to the reversible hydrogen electrode (RHE) scale, which was calculated by

$$E_{\mathrm{RHE}}=E_{\mathrm{Ag}/\mathrm{AgCl}}+0.199\;V+0.059\;V\cdot \mathrm{pH}$$

### 2.3 Product Quantification With Gas Chromatography

Potentiostatic electrolysis experiments coupled with online gas chromatography analysis were conducted in a standard H‐type cell separated by a Nafion 115 membrane (Fuel Cell Store). The anodic compartment utilized a carbon rod CE in 0.01 M H_2_SO_4_, while the cathodic compartment housed the WE and the RE in the respective 0.1 M K_2_SO_4_ electrolytes with pH 3.0, 2.5, and 2.0. To assess the influence of hydrodynamic modulation on mass transport, the CO_2_ purging intensity was varied between a low convection baseline (3 mL/min, inlet distal to the catalyst) and a high convection condition (10 mL/min, inlet positioned proximal to the catalyst surface). Each applied potential was maintained for 55 min, yielding H_2_ and FA as the predominant products. CO was detected in negligible quantities (<1%) and was consequently excluded from further analysis. Prior to each potential step, an oxidation cycle was performed to regenerate the catalyst's native surface oxide. As established in our previous publication [18], the presence of this surface oxide is critical for enhanced CO_2_RR performance, and this procedure ensures that the initial, highly active state is successfully regained for each measurement. Although this oxide layer gradually reduces under cathodic conditions, it remains sufficiently stable to provide a consistent catalytic interface throughout the 55 min duration of each potential step.

Gaseous products were quantified using a gas chromatograph (GC; NEXIS GC‐2030, Shimadzu) equipped with a ShinCarbon ST 100/20 column (2 m, 1 mm ID, 1/16 OD, Silco) for separation, employing a thermal conductivity detector (TCD) and a flame ionization detector (FID) for detection. The FE for gaseous species was calculated based on the average of the five GC measurements taken during each applied potential according to Equation (1):

(1) $$\mathrm{FE}\left(\%\right)=\frac{I_{\text{product}}}{I_{\text{total}}}=\frac{10^{-6}\cdot {\mathrm{ppm}}_{\text{product}}\cdot \frac{p\cdot \emptyset_{V}}{R\cdot T}\cdot z\cdot F}{I_{\text{total}}}\cdot 100$$

with ppm_product_ being the concentration of the product determined by GC (ppm), *p* the system pressure in the GC (bar), $\emptyset_{V}$ the volumetric gas flow rate (L/s), *R* the ideal gas constant (L bar/mol K), *T* the absolute temperature (K), *z* the number of electrons transferred per mol of product, *F* the Faraday constant (A s/mol), *I*_total_ the total current (A), and *I*_product_ the partial current (A) for each product.

Liquid products, specifically FA, were quantified via a gas chromatograph‐mass spectrometer (GC–MS; NEXIS GC‐2030 coupled to a QP 2020 NX, Shimadzu). To overcome the low volatility of FA and enhance detection sensitivity, the samples underwent derivatization to ethyl formate prior to analysis using a Shimadzu HS‐20 NX headspace sampler. Chromatographic separation was performed on a mid‐polarity SH‐I‐624Sil MS column (20 m, 0.18 mm ID, 1.0 µm film thickness). The FE for FA was determined from its total accumulation over the duration of the applied potential according to Equation (2):

(2) $$\mathrm{FE}\left(\%\right)=\frac{m_{\text{measured}}}{m_{\text{calculated}}}=\frac{c_{\text{product}}\cdot V_{\text{catholyte}}}{\frac{I\cdot t}{F\cdot z}\cdot M_{\text{product}}}\cdot 100$$

with *m* being the calculated or measured mass of the product (g), *c*_product_ the concentration of the product (g/L), *V*_catholyte_ the volume of the catholyte solution (L), *I* the total current (A), *t* the time (s), *F* the Faraday constant (A s/mol), *z* the number of electrons transferred per mol of products, and *M* the molar mass of the product (g/mol).

The produced amount of H_2_ and FA was calculated as follows.

(3) $$n_{H_{2}}=\frac{I\cdot t}{F\cdot z}\cdot {\mathrm{FE}}_{H_{2}}$$

(4) $$n_{\mathrm{FA}}=\frac{c_{\mathrm{FA}}\cdot V_{\text{catholyte}}}{M_{\mathrm{FA}}}$$

with *n* being the amount of H_2_ and FA (mol).

## 3 Results and Discussion

### 3.1 Electrochemical Response and Product Distribution as a Function of pH

It is well established that the HER in a pH range between 2 and 5 bifurcates into distinct diffusion‐limited proton and water reduction pathways [23, 24, 25]. We showed in our previous work [18] that, in mildly acidic media, the CO_2_RR to FA occurs in the potential window between these two processes, and we were able to achieve FEs to FA exceeding 90% on carbon‐supported Bi nanoparticles (Bi/C) at pH 3. In this study, we extend the investigation to more acidic conditions (pH 2.0 and 2.5) to systematically evaluate the influence of proton activity on the CO_2_RR. Acidified potassium sulfate (K_2_SO_4_) was employed as the supporting electrolyte to leverage the dual benefits of K^+^ cations, which stabilize critical CO_2_ reduction intermediates through local field effects, and sulfate anions, whose specific adsorption aids in suppressing the competing HER without altering the CO_2_RR selectivity pathway [22, 26, 27, 28].

**FIGURE 1:**
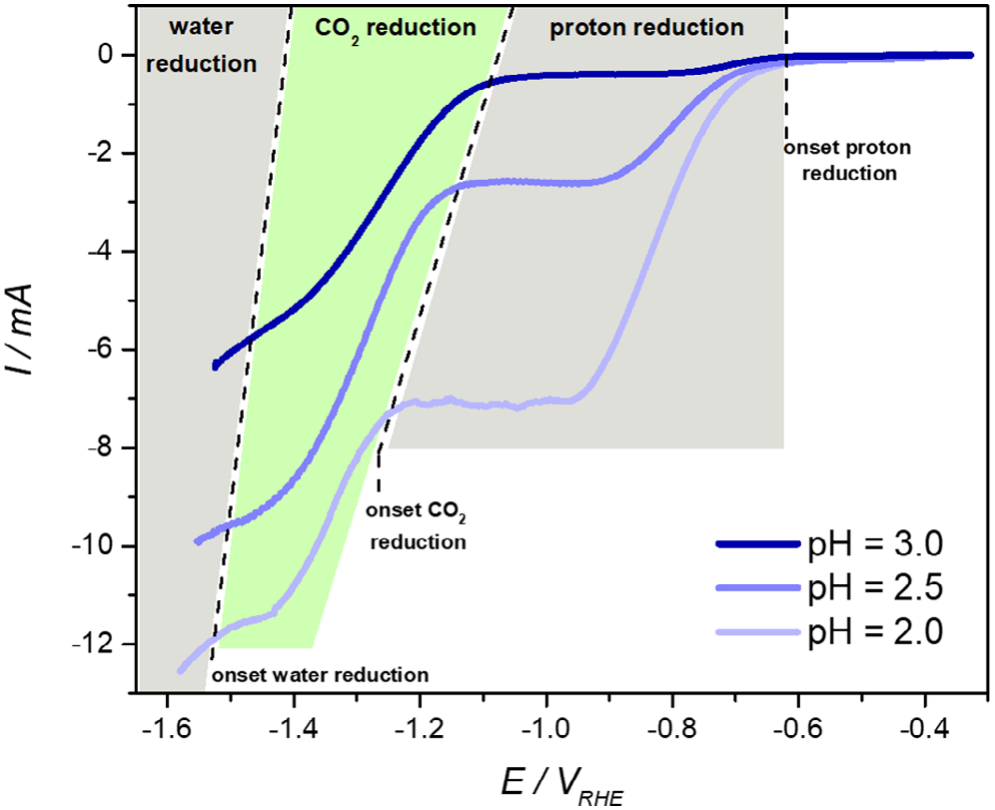
Linear sweep voltammetry (LSV) plots at pH 3.0, 2.5, and 2.0, with Bi/C in CO_2_‐saturated acidified 0.1 M K_2_SO_4_. Scan rate: 50 mV/s. The diffusion‐limited current for proton reduction increases notably with decreasing pH, directly correlating with the higher bulk proton concentration. While the onset potential for proton reduction remains invariant, the onset of the CO_2_RR exhibits a cathodic shift from ~−1.1 *V*_RHE_ at pH 3.0 to ~−1.3 *V*_RHE_ at pH 2.0.

**FIGURE 2:**
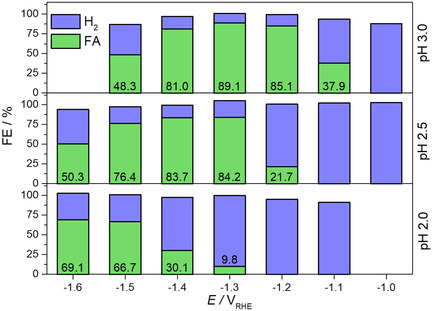
Potential‐dependent Faraday efficiency (FE) profiles of the primary products, formic acid (FA), and H_2_, at pH 3.0, 2.5, and 2.0, recorded under low convection conditions (CO_2_ flow rate: 3 mL/min; compare experimental section) with Bi/C in CO_2_‐saturated 0.1 M K_2_SO_4_. The data shows a decrease in maximum FA selectivity from ~ 89% at pH 3.0 to ~ 69% at pH 2.0, with a shift of the maxima from −1.3 to −1.6 *V*_RHE_. The onset potential for the CO_2_RR shows a similar cathodic shift from −1.1 to −1.3 *V*_RHE_.

Initially, we examined the electrochemical response via LSV (Figure 1) across three distinct pH levels with a typical three‐electrode setup. The current in the first reduction region increases markedly with decreasing pH. As proven by our GC product analysis (Figure 2), this elevated current is exclusively attributed to H_2_ evolution, evidenced by a FE for H_2_ of nearly 100% in this regime. This is further confirmed by the Ar‐purged LSVs (Figure S1), where exclusively H_2_ is formed, demonstrating the exact same diffusion‐limited behavior. Consequently, these data directly confirm that a lower bulk pH significantly increases the interfacial proton flux to the catalyst surface. Furthermore, the onset potential for the CO_2_RR exhibits a cathodic shift from ~−1.1 *V*_RHE_ at pH 3.0 to ~−1.3 *V*_RHE_ at pH 2.0. This negative shift is mainly attributable to kinetic suppression of the CO_2_RR driven by the enhanced proton activity, which likely induces increased proton surface excess at the WE and activation inhibition of the CO_2_ reduction. Similar to the trend observed for CO_2_ reduction, the onset of water reduction also shifts to more negative potentials with decreasing pH. A similar shift occurs in Ar‐purged electrolyte (Figure S1); however, water reduction onset starts at lower cathodic potentials compared to CO_2_‐saturated conditions, indicating that the CO_2_RR effectively suppresses water reduction. In contrast, the onset for the proton reduction itself shows classical Nernstian behavior; i.e., it does not shift with decreasing pH on the RHE scale.

To evaluate the potential‐dependent selectivity of the catalyst, potentiostatic electrolysis was performed in a H‐cell setup across the investigated pH range at potentials ranging from −1.0 to −1.6 *V*_RHE_, in 0.1 V steps (see Experimental Section in the Supporting Information for more details). Figure 2 illustrates the pH‐dependent product distribution, which corroborate the electrochemical trends observed in the LSVs (Figure 1). Notably, the onset potentials for FA production correlate well with the CO_2_RR onset potentials determined via LSV. A distinct trend is evident wherein the selectivity toward FA diminishes as the pH decreases, driven by the competitive rise in proton reduction at higher proton concentrations. Concomitantly, the total current increases with decreasing pH (Figure S2). Quantitatively, the system achieves a FE of approximately 89% at pH 3.0, compared to only ~69% at pH 2.0. Consequently, based on selectivity and onset potential metrics alone, pH 3.0 appears to be the optimal pH for CO_2_RR to FA. Although higher pH values up to 4.25 (corresponding to the native pH of CO_2_‐saturated 0.1 M K_2_SO_4_, Figure S3) exhibit comparable selectivity, they were excluded from further study due to temporal instability. Specifically, the bulk pH in these unbuffered solutions was found to rapidly drift toward more acidic pH values during operation, preventing the maintenance of a steady‐state reaction environment.

At pH 3.0, the potential step series was terminated at −1.5 *V*_RHE_ due to the onset of water reduction (Figure 1). Water reduction initiates at lower overpotentials at pH 3.0 compared to the more acidic electrolytes, likely due to the reduced FA formation ability at the catalyst surface, which leaves a greater density of surface sites available for water activation. Once initiated, water reduction induces a significant local pH shift that fundamentally alters the reaction environment [23]. This effect, which leads to a strong increase in current over time (Figure S2), has been characterized in our previous work [18]. Conversely, at pH 2.0, the potential step at −1.0 *V*_RHE_ was omitted, as the onset of FA formation in this pH regime does not occur until −1.3 *V*_RHE_.

### 3.2 Relation Between Proton Activity and Formic Acid Production

To holistically assess electrolyzer performance, the analysis should encompass not only selectivity and onset potential determination but also include the identification of partial currents or current densities, which directly govern the product formation rate. While pH 3.0 appears optimal in terms of onset potential and FE, an assessment of the production rate reveals a different result. Despite the more negative onset potential and reduced selectivity observed at pH 2.0, the absolute yield of FA is significantly enhanced at this lower pH regime.

**FIGURE 3:**
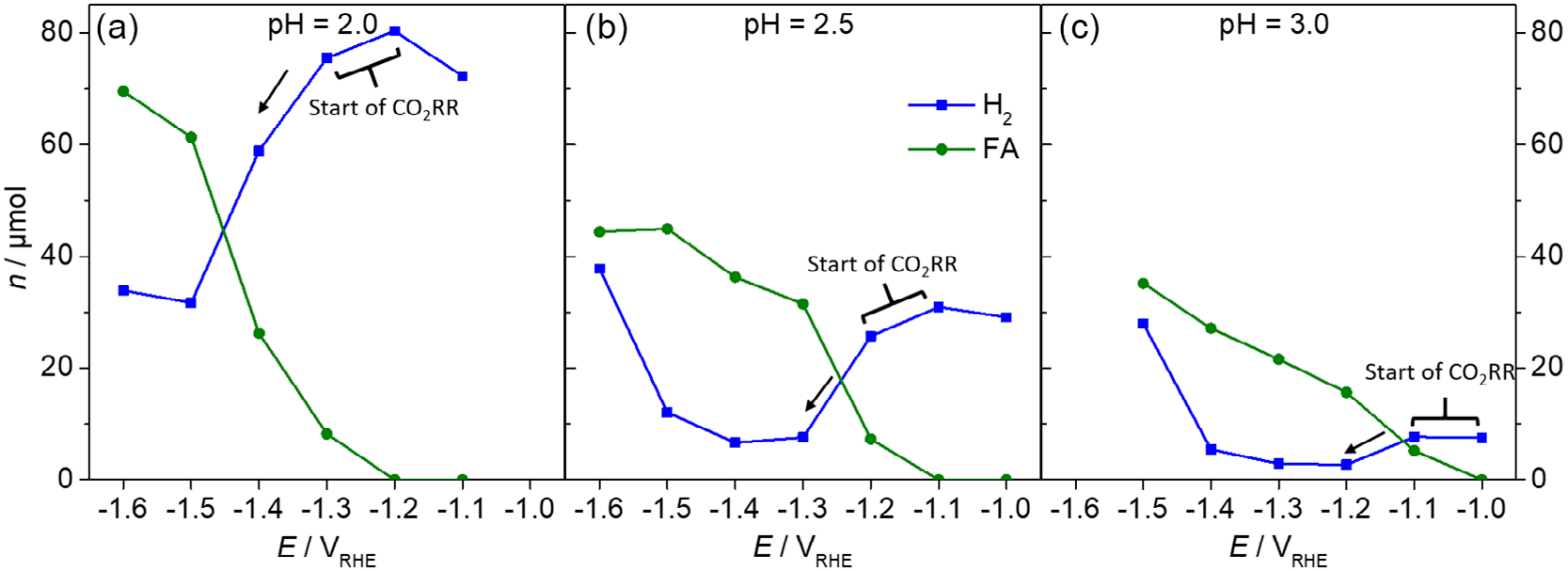
Potential‐dependent amounts of H_2_ (blue) and FA (green) obtained by potentiostatic electrolysis at pH 2.0 (a), 2.5 (b), and 3.0 (c), recorded under low convection conditions (CO_2_ flow rate: 3 mL/min) with Bi/C in CO_2_‐saturated 0.1 M K_2_SO_4_. While the absolute amount of H_2_ increases with decreasing pH, it is suppressed upon the onset of the CO_2_RR at each pH value. The highest production rate of FA is achieved at pH 2.0, which shows that a higher proton availability at the surface is beneficial for the FA formation.

Figure 3 elucidates this interesting finding through plots of the specific product formation rates for the different pH values and applied potentials. Consistent with the higher bulk proton concentration, the amount of H_2_ increases with decreasing pH; however, a distinct suppression of H_2_ formation is observed concomitant with the onset of the CO_2_RR for all tested pH values, validating the competitive consumption of interfacial protons.

At low cathodic overpotentials (−1.1 *V*_RHE_), measurable FA formation is restricted to pH 3.0. As the potential becomes more cathodic, the optimal pH for maximizing FA production shifts. While pH 3.0 is favorable initially, pH 2.5 generates the highest amount of FA at −1.3 *V*_RHE_ and pH 2.0 surpasses the others at −1.5 *V*_RHE_. Notably, while pH 3.0 reaches superior selectivity, the FA production rate at pH 2.0 at −1.6 *V*_RHE_ is nearly double of that observed at pH 3.0. This highlights that the enhanced total current (Figure S2) at lower pH is not solely attributable to H_2_ evolution but rather drives also a marked increase in FA production rates. These findings clearly show that elevated interfacial proton availability is beneficial for FA synthesis, as protons are essential for the FA formation (see Equation I).

To substantiate the hypothesis that proton activity constitutes the rate‐limiting factor at pH 3.0, we calculated the theoretical diffusion‐limited current densities ($j_{\lim}$) for both the proton reduction reaction and the CO_2_RR across the investigated pH range. In this analysis, the bulk CO_2_ concentration is assumed to remain constant at saturation, whereas the bulk proton concentration varies inversely with pH. The theoretical mass transport limiting currents were derived using the following mass transport equations [29]:

(II) $$j_{\lim,H^{+}}=z\cdot F\cdot D_{H^{+}}\cdot \frac{c_{H^{+}}}{\delta }$$

(III) $$j_{\lim,{\mathrm{CO}}_{2}}=z\cdot F\cdot D_{{\mathrm{CO}}_{2}}\cdot \frac{c_{{\mathrm{CO}}_{2}}}{\delta }$$

with *j*_lim_ being the theoretical diffusion‐limited current caused by H^+^ or CO_2_ reduction (mA/cm^2^), *z* the number of electrons transferred, *F* the Faraday constant (C/mol), *D* the diffusion coefficient of H^+^ (9.3 · 10^−5^ cm^2^/s) [30] or CO_2_ (1.9 · 10^−5^ cm^2^/s) [31], *c* the concentration of H^+^ (10^−pH^ mol/L) or CO_2_ (3.4 · 10^−2^ mol/L) [32] in the solution, and *δ* the thickness of the diffusion boundary layer (cm).

While the diffusion boundary layer thickness remains an experimentally variable parameter, it is assumed to be equivalent for both species under constant hydrodynamic conditions [29]. Consequently, the layer thickness (*δ*), the number of electrons (*z*), and the Faraday constant (*F*) cancel out, which allows for a direct assessment of the relative mass transport limitations via the ratio of the theoretical diffusion‐limited current densities:

(IV) $$\text{Ratio}=\frac{j_{\lim,H^{+}}}{j_{\lim,{\mathrm{CO}}_{2}}}=\frac{D_{H^{+}}\cdot c_{H^{+}}}{D_{{\mathrm{CO}}_{2}}\cdot c_{{\mathrm{CO}}_{2}}}$$

At pH 3.0, this ratio is calculated as 0.14, highlighting that the transport of CO_2_ to the surface significantly exceeds the flux of protons under mass transport–limited conditions. This identifies the proton supply as the rate‐limiting factor at pH 3.0. At pH 2.5, the ratio increases to 0.46 due to the elevated bulk proton concentration, yet the electrocatalytic performance of the system remains proton‐limited. At pH 2.0, a fundamental shift in the mass transport regime occurs, because the ratio rises to 1.44 and the proton flux exceeds the CO_2_ supply. Under these conditions, the stoichiometric excess of interfacial protons accelerates the parasitic HER, which rationalizes the decrease in FE toward FA observed in Figure 2. However, this elevated proton availability simultaneously accelerates the kinetics of FA formation (Equation I), which explains the measured increase in FA production rate with decreasing pH (Figure 3). It is important to note that while the local pH at the catalyst interface inherently deviates from the bulk value during active electrolysis, the maximum interfacial proton flux remains fundamentally governed by the bulk proton concentration. Consequently, a lower bulk pH systematically increases the mass transport–limited proton supply to the active sites, driving the observed kinetic enhancements.

To experimentally decouple the effects of proton activity and mass transport on FA formation kinetics, two methodological approaches are possible. The first involves elevating the CO_2_ partial pressure within the cathode compartment to determine if increasing the concentration of dissolved CO_2_ at pH 2.0 leads to an increase in selectivity for FA again. Alternatively, modulating the hydrodynamic conditions, specifically decreasing the diffusion boundary layer thickness (*δ*) through increased CO_2_ flow rates, offers a direct way to modify mass transport limitations. A reduction in *δ* is predicted to enhance the proton diffusion–limited current at pH 3.0 [29], which leads to an increase of the proton activity at the catalyst surface. Due to experimental setup constraints precluding high‐pressure operation, we pursued the hydrodynamic modulation approach. We reduced the diffusion boundary layer thickness (*δ*) by increasing the CO_2_ flow rate from 3 to 10 mL/min (from low to high convection conditions) and orienting the CO_2_ gas inlet in the immediate vicinity of the catalyst surface to maximize turbulence.

**FIGURE 4:**
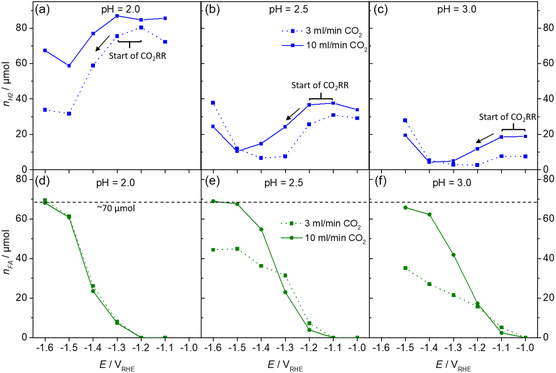
Potential‐dependent product formation rates derived from potentiostatic electrolysis at pH 2.0, 2.5, and 3.0, recorded under high convection conditions (solid lines, CO_2_ flow rate: 10 mL/min) with Bi/C in CO_2_‐saturated 0.1 M K_2_SO_4_. For comparison, the low convection conditions (3 mL/min CO_2_) from Figure 3 are inserted as dotted lines. (a–c) Formation rates of H_2_, showing the increase in the HER compared to the low convection conditions, confirming enhanced proton mass transport. (d–f) Formation rates of FA, demonstrating that enhanced hydrodynamic conditions augment FA formation by increasing interfacial proton activity for pH of 3.0 and 2.5. At pH 2.0, the formation rate of FA remains invariant, due to sufficient proton supply at the surface.

Figure 4a–c illustrates the amount of H_2_ formed under these enhanced hydrodynamic conditions. The H_2_ production observed at elevated CO_2_ flow rates (solid lines) is augmented compared to the low CO_2_ flow rates (dotted lines), confirming the successful increase of proton mass transport to the electrode surface. This trend is corroborated by the total Faradaic currents, which exhibit a corresponding increase under high convection conditions (Figure S4), compared to the low convection conditions (Figure S2). Consistent with previous observations, the HER is suppressed concomitant with the onset of the CO_2_RR, evidenced by the decrease in the amount of H_2_ formed.

Figure 4d–f compares FA formation rates under high (solid lines) versus low (dashed lines) CO_2_ flow rates. At pH 3.0 and 2.5, high convection conditions increase FA production noticeably, which convincingly validates the critical role of proton availability for this reaction. Although high FA selectivity is maintained under high convection (Figure S5), the maximum FE occurs at a ~0.1 V more cathodic potential compared to the low convection regime (Figure 2) due to intensified HER competition. Nevertheless, once this kinetic hurdle is overcome at higher overpotentials, the increased interfacial proton supply facilitates substantially higher FA production rates. In contrast, FA formation at pH 2.0 (Figure 4d) remains invariant, indicating that the interfacial proton activity is already sufficient to saturate reaction sites. The transition to kinetic control is confirmed by the universal plateau for the amount of FA at ~70 µmol across all pH levels (see Figure 4d–f), indicating an intrinsic catalyst turnover ceiling. Theoretically, because hydrodynamic modulation enhances the mass transport of both protons and CO_2_, a transport‐limited system should respond with production rates proportional to the increased mass transport. However, the observed plateau at around 70 µmol demonstrates that reactant supply is no longer the rate‐determining factor. This is further proven by an experiment, where the catalyst loading was doubled (Figure S6), which resulted in an increased FA formation rate.

Importantly, these hydrodynamic modulation experiments (Figure 4) also confirm that the observed performance enhancements are fundamentally driven by mass transport rather than potential pH‐induced structural changes to the catalyst. By demonstrating that the FA formation rate increases significantly at a constant bulk pH solely through enhanced convection, we can definitively attribute the performance variation to the dynamic supply of protons rather than a static, pH‐dependent surface reconstruction or corrosion process.

## 4 Conclusion

This study provides a critical reassessment of the role of proton activity in the electrochemical reduction of CO_2_ to FA in acidic media, countering the traditional perspective that regards protons predominantly as parasitic agents. While suppressing the HER remains a primary objective in the acidic CO_2_RR, our results demonstrate that a simple “proton depletion” strategy, commonly effective for CO production, is the wrong methodology for FA electrosynthesis [4, 20, 21]. Our combined electrochemical and GC analysis reveals that the kinetics of FA formation are intimately coupled to interfacial proton availability. We observe that decreasing the electrolyte pH naturally increases the parasitic HER, but it simultaneously enhances the FA production rates, despite a reduction of the FE toward FA formation. Moreover, the highest FA amount is measured at pH 2.0, where the proton supply is abundant, rather than at higher pH levels, where the reaction becomes mass transport–limited due to a decreased proton activity in the electrolyte.

Collectively, these findings establish that high interfacial proton activity is an indispensable kinetic driver for FA synthesis. Consequently, future design strategies must move beyond simple HER suppression and instead focus on kinetic balancing through the supply of a sufficiently high proton flux, while simultaneously maximizing the local CO_2_ concentration, to enable high FA formation rates and selectivities potentially through pressurized systems or advanced gas diffusion architectures. This paradigm shift offers a viable pathway for scaling up acidic FA production by leveraging the beneficial role of protons rather than treating them solely as a liability.

## Author Contributions

J.K.‐L. and A.B. supervised and coordinated this work. T.M. designed and conducted all the LSV and GC experiments. T.M., C.G., A.B., P.S., and J.K.‐L contributed to the manuscript writing. All authors discussed and revised the manuscript.

## Funding

This study was supported by the Austrian Science Fund (grant 10.55776/COE5).

## Conflicts of Interest

The authors declare no conflicts of interest.

## Supporting information

Experimental section, LSV plots in Ar‐purged electrolyte, FE for the pH values of 3.5 and 4.25, potential‐dependent current responses at low and high convection conditions, FE at high convection conditions, and FA production rate for different catalyst loadings.

## Data Availability

The data that support the findings of this study are openly available in InvenioRDM at https://doi.org/10.48323/aes48‐v7z39.
