# Supplementary material for: Proton Activity as a Promoter for the CO2 Electroreduction to Formic Acid in Acidic Media
Source: ChemSusChem. 2026 Jul 23;19(15):e70906. doi: 10.1002/cssc.70906 (PMC13393336; doi:10.1002/cssc.70906)
Supplement: Supplementary file 1 — Experimental section, LSV plots in Ar‐purged electrolyte, FE for the pH values of 3.5 and 4.25, potential‐dependent current responses at low and high convection conditions, FE at high convection conditions, and FA production rate for different catalyst loadings. [file CSSC-19-e70906-s001.pdf]

Supplementary Information

Proton Activity as a Promoter for the CO_2 Electroreduction to Formic Acid in Acidic Media

Thomas Mairegger,[1,2] Christoph Griesser,[1] Philipp Stadler,[2] Alexander Beck,[2] and Julia Kunze-Liebhäuser[1]*

[1]Department of Physical Chemistry, University of Innsbruck, Innrain 52c, 6020-Innsbruck, Austria.
[2]Net Zero Emission Labs GmbH, Sinning 1, 83101-Rohrdorf, Germany

*Corresponding author: Julia.Kunze@uibk.ac.at

This PDF file includes:

Supplementary figures

Supplementary Figure 1. Linear sweep voltammetry plots in Ar purged electrolyte

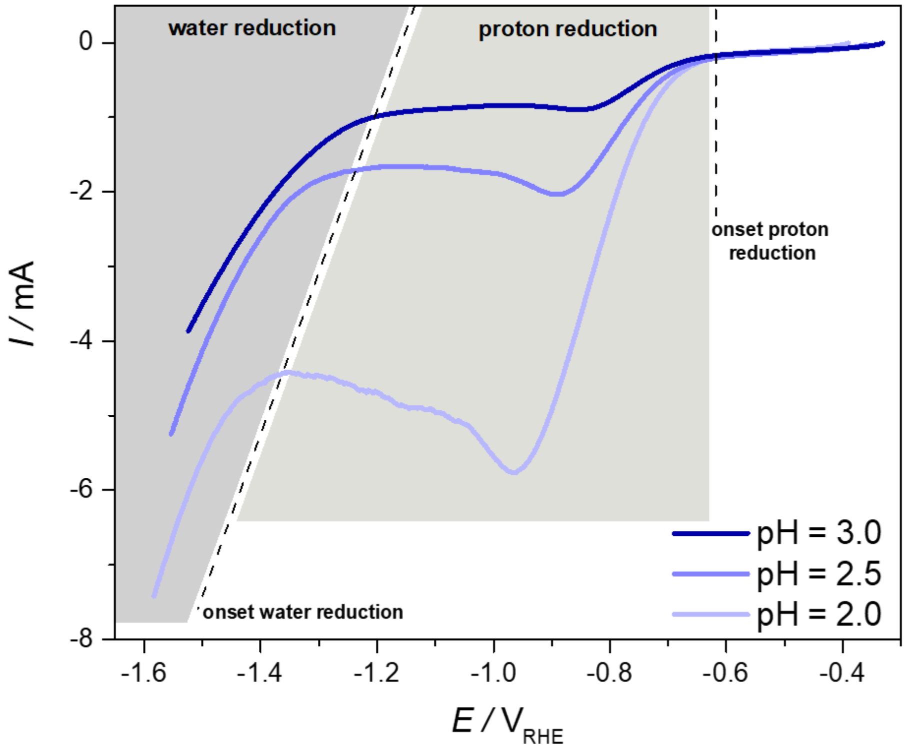

Figure S1: Linear sweep voltammetry (LSVs) plots at pH 3.0, 2.5 and 2.0, with Bi/C in Ar purged acidified 0.1 M K_2SO_4. Scan rate: 50 mV/s. Consistent with Figure 1, the onset potential for proton reduction remains invariant, while the onset for the water reduction shifts with lower pH due to the increased proton concentration in the electrolyte. A comparison with Figure 1 reveals that water reduction is notably suppressed in the CO_2-saturated electrolyte, indicating that the CO_2RR competitively inhibits the water reduction.

Supplementary Figure 2. Potentiostatic electrolysis at low convection conditions

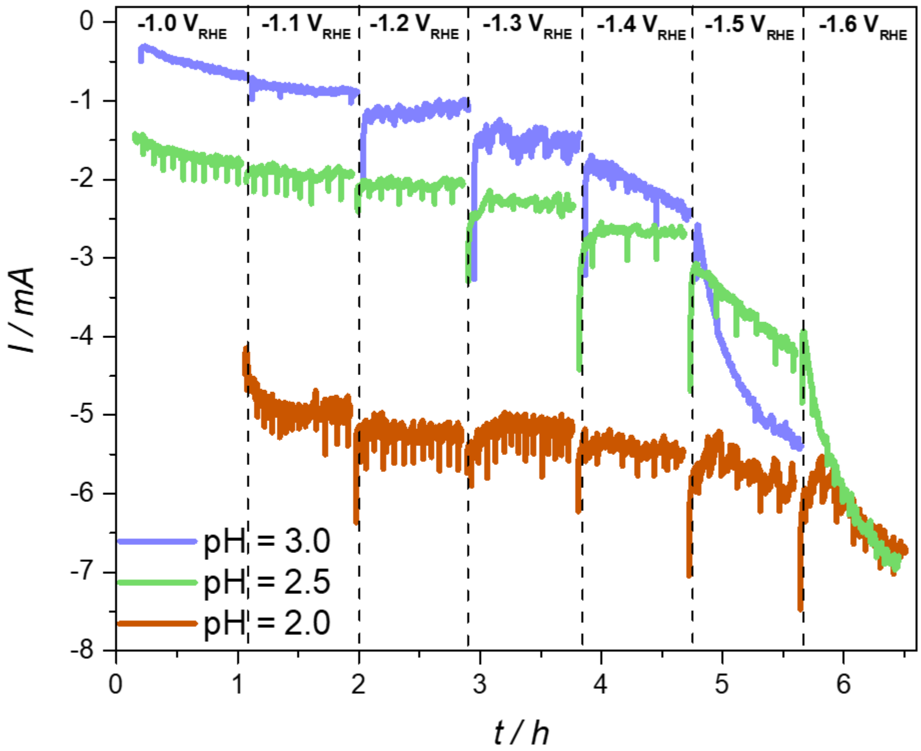

Figure S2: Potential-dependent current response at pH 3.0, 2.5 and 2.0, recorded under low convection conditions (CO_2 flow rate: 3 ml/min) with Bi/C catalyst in CO_2-saturated 0.1 M K_2SO_4. The magnitude of the total current increases inversely with pH, driven by the higher bulk proton concentration. Starting at -1.5 V_{RHE}, the current increases heavily over time for pH 3.0 and, to a lesser extent, for pH 2.5, which indicates the water reduction onset. At pH 2.0, the onset of the water reduction is shifted to -1.6 V_{RHE} and the increase is attenuated, due to the high proton concentration.

Supplementary Figure 3. FE for potentiostatic electrolysis at pH 3.5 and 4.25 at low convection conditions

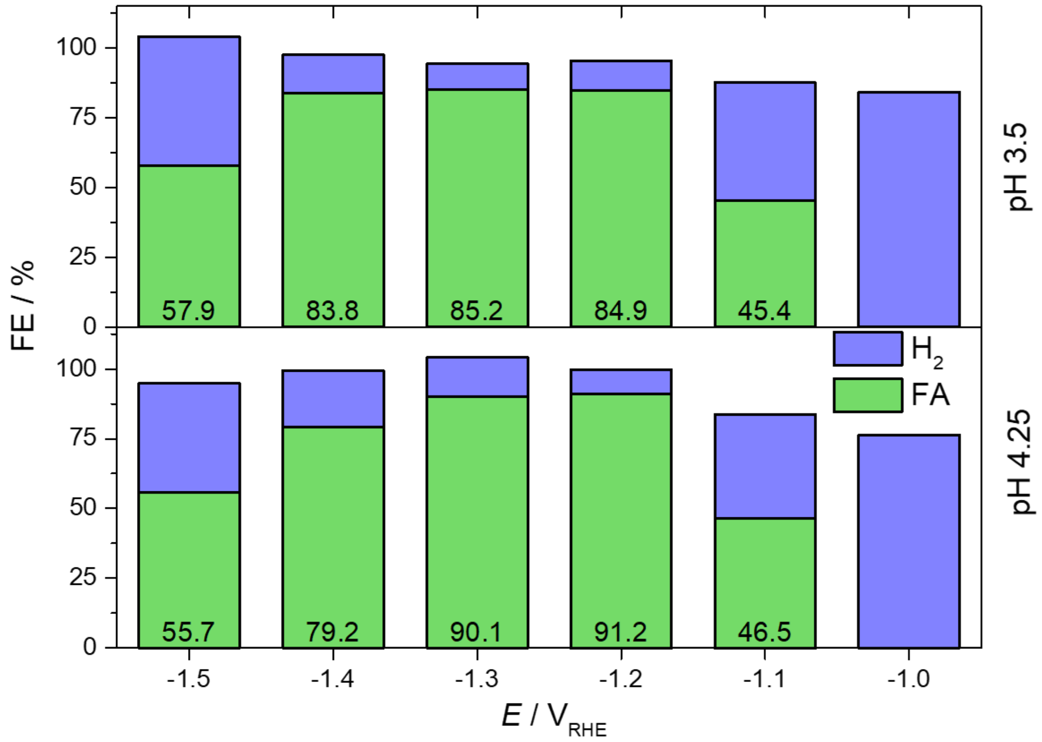

Figure S3: Potential-dependent FE profiles of the primary products, formic acid (FA) and H_2 at pH 3.5 and 4.25 (corresponding to the native pH of CO_2-saturated 0.1 M K_2SO_4), recorded under low convection conditions (CO_2 flow rate: 3 ml/min) with a Bi/C catalyst in CO_2-saturated 0.1 M K_2SO_4. While the product selectivity is similar to that observed at pH 3.0 (Figure 1), these pH values were excluded from further study due to the rapid acidification of the bulk electrolyte, which prevented the maintenance of a stable reaction environment.

Supplementary Figure 4. Potentiostatic electrolysis at high convection conditions

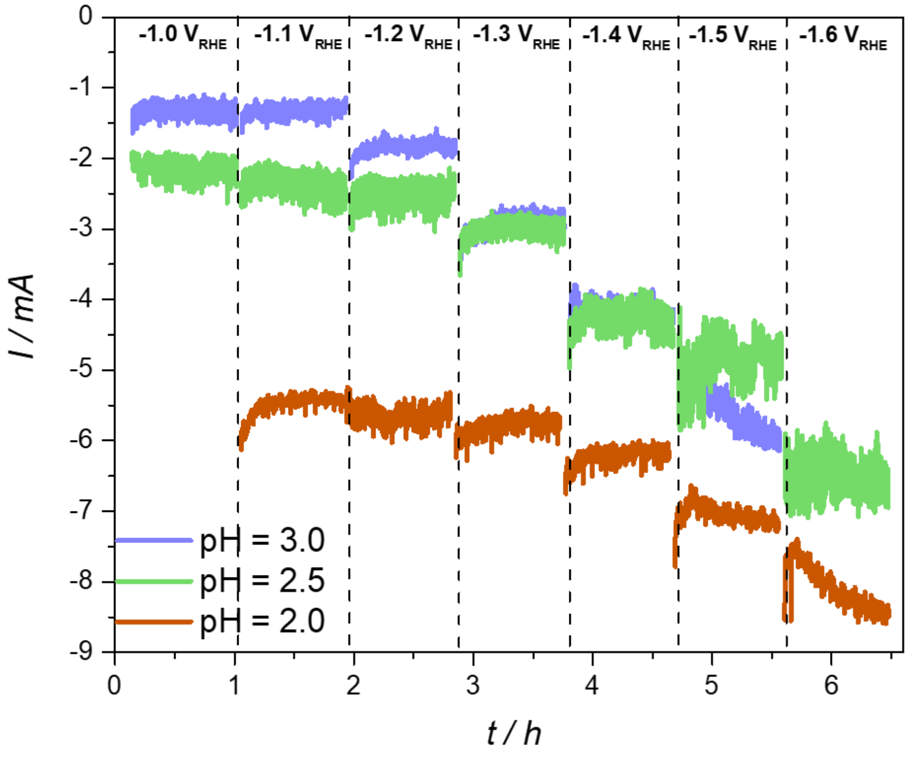

Figure S4: Potential-dependent current response at pH 3.0, 2.5 and 2.0, recorded under high convection conditions (CO_2 flow rate: 10 ml/min) with a Bi/C catalyst in CO_2-saturated 0.1 M K_2SO_4. Analogous to Figure S1 the total current scales inversely with pH. However, a comparative increase in current is observed, which confirms that the increased flow rate effectively enhances proton mass transport. Following the onset of the CO_2RR (-1.1 V_{RHE} at pH 3.0 and -1.2 V_{RHE} at pH 2.5), the current increments between consecutive potential steps are notably magnified relative to the trends observed in Figure S2, signifying enhanced CO_2RR currents.

Supplementary Figure 5. FE for potentiostatic electrolysis at high convection conditions

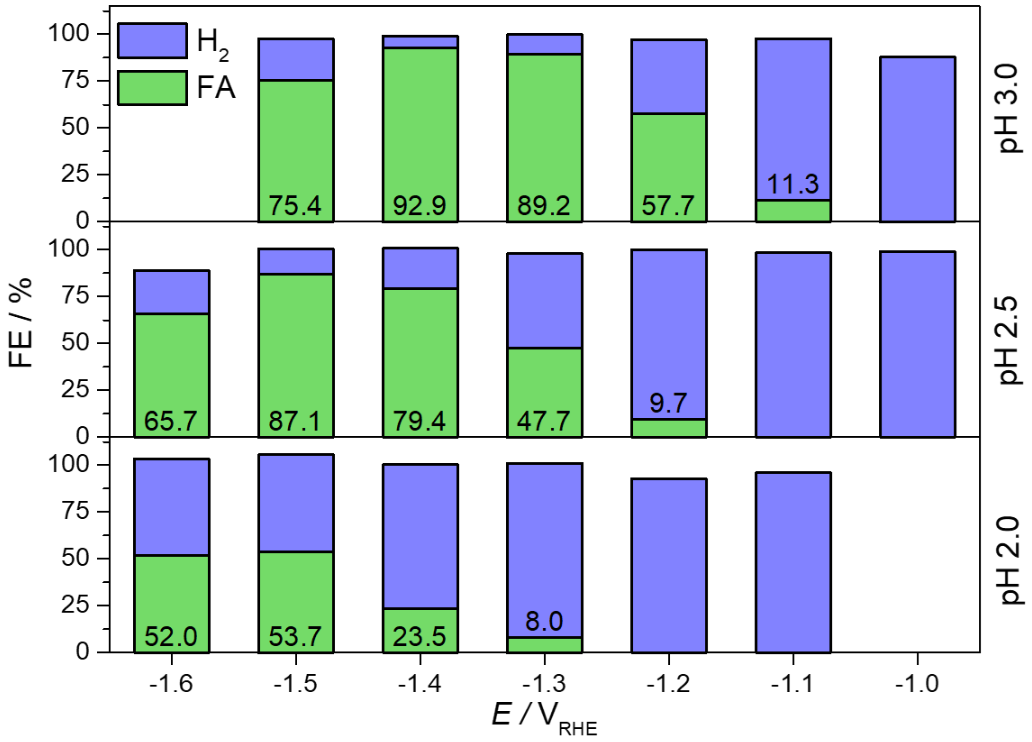

Figure S5: Potential-dependent Faraday efficiency (FE) profiles of the primary products, FA and H_2, at pH 3.0, 2.5 and 2.0, recorded under high convection conditions (CO_2 flow rate: 10 ml/min) with a Bi/C catalyst in CO_2-saturated 0.1 M K_2SO_4. The selectivity trends mirror those observed under the low convection conditions in Figure 2, demonstrating that hydrodynamic modulation does not significantly change the product distribution. However, together with the total currents plotted in Figure S4, the data confirm that the increased proton flux directly accelerates the kinetics of the FA production without compromising selectivity.

Supplementary Figure 6. Amount of FA formed with different catalyst loadings

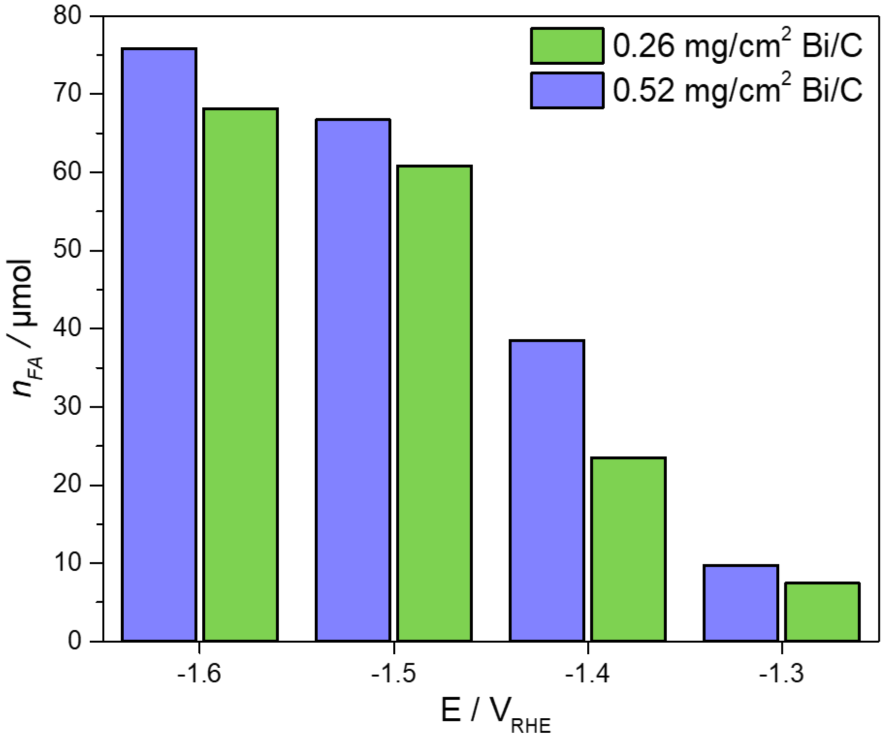

Figure S6: Potential-dependent amounts of FA at a pH of 2.0, recorded under high convection conditions (CO_2 flow rate: 10 ml/min) with different Bi/C catalyst loadings in CO_2-saturated 0.1 M K_2SO_4. While increasing the catalyst loading enhances the absolute amount of FA produced, the improvement is not proportional to the increase in mass. This sub-linear scaling is likely attributable to the physical structure of the drop-casted film and the high Nafion content (30 wt%); as the catalyst layer thickens, the underlying active sites become increasingly inaccessible to reactants, resulting in diminished catalyst utilization efficiency.
